# The Association between Executive Function and Performing Instrumental Daily Activities in People with Intellectual Disabilities

**DOI:** 10.3390/healthcare11172374

**Published:** 2023-08-23

**Authors:** Beatriz García-Pintor, Francisco Manuel Morales-Rodríguez, José Manuel Pérez-Mármol

**Affiliations:** 1 Association in Favour of People with Intellectual Disability—ASPROGRADES, 18007 Granada, Spain; beatrizpintor@gmail.com; 2 Department of Physiotherapy, Faculty of Health Sciences, University of Granada, 18071 Granada, Spain; 3 Department of Educational and Developmental Psychology, Faculty of Psychology, University of Granada, 18011 Granada, Spain; fmmorales@ugr.es; 4 Instituto de Investigación Biosanitaria ibs.GRANADA, 18012 Granada, Spain

**Keywords:** intellectual disability, instrumental activities of daily living, IADLs, function, functionality, executive function, rehabilitation, occupational therapy

## Abstract

Institutionalized individuals with intellectual disabilities have few opportunities to participate in instrumental activities of daily living (IADLs), which probably affects higher cognitive functions, or vice versa. The objectives of this study were to evaluate the possible difference in the ability to perform IADLs and executive functioning between individuals with and without intellectual disabilities and to determine if executive functions are associated with the performance of IADLs in people with intellectual disabilities. This was a multi-center cross-sectional study, conducted between July 2019 and May 2020. Participants with intellectual disabilities were recruited from four centers for people with intellectual disabilities. Adults without these disabilities were gathered from several community centers. The sample consisted of 90 individuals with moderate intellectual disabilities and 79 individuals with no intellectual disability. Executive functions were evaluated using the Wechsler Adult Intelligence Scale—WAIS-IV, the INECO Frontal Screening test, the Semantic Verbal Fluency Test, and the Behavioural Assessment of the Dysexecutive Syndrome—BADS—Scale. The performance of the IADLs was assessed by the Lawton and Brody Scale. The results showed that the higher the function in instrumental activities, the lower the impairment of executive functions. Executive functions accounted for 81% of the total variance in the ability to perform the IADLs. In conclusion, individuals with moderate intellectual disabilities demonstrated limitations in executing the IADLs, which were partially associated with low performance in executive functions. This information could help in the development of evidence-based intervention programs and facilitate the formulation of appropriate support strategies to enhance participation in these activities.

## 1. Introduction

The functional abilities—or functionality—of individuals with intellectual disability (ID) are currently understood from a multidimensional perspective. According to the International Classification of Functioning, Disability, and Health, functionality encompasses the activities, daily tasks, and abilities present throughout the individual’s life [1,2]. A functional limitation in daily tasks usually involves a disability stemming from problems related to body functions or structures [3]. Consequently, participation in activities of daily living throughout life generates the opportunity to develop effective functional, cognitive, and/or social motor skills. Activities of daily living can be divided into basic and instrumental activities. The performance of basic activities involves simpler skills. These activities are focused on self-care such as showering, dressing, grooming, or eating. Instrumental activities of daily living (IADLs) require more complex skills that are usually executed in interaction with the social environment such as caring for other people, mobility in the community, the use of new technologies, or management of one’s own finances [3]. People with ID do not always have the skills required to adequately perform the more complex activities of daily living. Hence, the number of opportunities for people with ID to participate in activities of daily living may be limited in diverse contexts such as family or institutional life [4]. To the best of our knowledge, no other studies have evaluated the level of function with respect to performing these activities in ID populations. Only one other study has evaluated functionality in living skills, without focusing specifically on IADLs [5]. The functional capacity of these people could be improved by providing them with support in the performance of these activities, enabling and maximizing their own abilities [6]. To do this, additional studies are required to determine what are the factors associated with functionality in activities of daily living in people with ID.

Higher cognitive functions may play a significant role among the potential factors related to functionality. As higher cognitive functions become impaired, individuals may experience challenges in planning and performing complex tasks, managing their time, and adapting to changes in their environment. Executive control, embodying higher cognitive function, is defined as the self-regulatory processes that facilitate the supervision, organization, and coordination of other cognitive functions as well as emotional and behavioral responses [7]. Although there are different classifications of executive functions, the most widely used constructs in research are action planning ability, working memory, inhibitory control, verbal fluency, or abstraction capacity [7]. Action planning ability refers to the skills to anticipate future events [8]. Working memory is the ability to organize, synthesize, relate, and use information in order to perform other more complex cognitive activities [9]. Inhibitory control is considered one of the basic mechanisms of adaptive behavior. This construct is involved in new or complex situations and scenarios in which integration of experience and knowledge is important [10]. Verbal fluency is recognized as the ability to produce language using inhibitory control and certain memory processes. For this reason, the verbal fluency may be an index of mental processing. This function is associated with cognitive processing and thought processes [8]. Abstraction consists of identifying the common elements in things that are apparently unconnected [11].

The potential reasons for the potential link between executive functions and the capacity of individuals with ID to effectively perform IADLs may be grounded: (i) in the executive processes that underpin the ability to plan, organize, prioritize, and adapt in response to the demands of daily life; (ii) deficits in executive functions could significantly impact an individual’s ability to perform IADLs, leading to challenges in everyday functioning; (iii) the functions such as planning, cognitive flexibility, inhibitory control, and working memory, are essential for successful completion of complex tasks and problem-solving. For instance, when an individual engages in activities such as cooking, managing finances, or organizing their schedule, they rely on their executive functions to plan and sequence the steps required to complete the task efficiently; (vi) these functions are vital for time management and decision-making, which are fundamental aspects of many IADLs. For instance, planning a daily routine, prioritizing tasks, and setting goals all require these cognitive processes; and (v) the ability to switch between different tasks and manage multiple activities simultaneously is dependent on cognitive flexibility. In the context of IADLs, this is particularly relevant as individuals frequently need to manage various responsibilities throughout the day [5,12,13,14,15,16,17,18]. However, to the best of our knowledge, no studies have evaluated if the performance of activities of daily living is related to these higher cognitive functions (executive functions) in institutionalized individuals with ID.

The literature highlights the importance of evaluating the magnitude of the association between these constructs in people with ID and points out participation in activities of daily living as a central aspect of human functioning [5,8,15,17,18]. A review indicated that further research is necessary to formulate a participation definition that harmonizes established descriptions from rehabilitation literature with aspects derived from ID studies [16]. Researchers such as Carretti et al. [17] have reported that understanding executive function is crucial to advancing patient-focused health models [17,18]. A study of patients with acquired frontal lobe injury has shown that individuals need executive functions, such as planning ability, self-correction, decision-making, and judgment, to correctly perform their daily activities [12]. In older adults, the common clinical measures of executive function are useful in predicting functional status [13,14]. A study of young adults with Down syndrome has suggested that executive function and adaptive behavior are associated with their employment status [15]. Therefore, it could be hypothesized that the successful performance of IADLs in other populations with impaired executive functions may also depend on the control of higher cognitive functions [5]. New studies should contribute to determining the factors that are associated with the performance of the activities of daily living in individuals with ID and the magnitude of this contribution. Therefore, the aims of the study were: to evaluate the possible difference in the ability to perform IADLs and executive functioning between individuals with and without ID and to determine if executive functions are associated with the performance of IADLs in people with ID.

## 2. Method

### 2.1. Design

This is a multicenter, cross-sectional study. The local Ethics Committee of CEI-Granada (Granada-Spain) approved the study protocol.

### 2.2. Participants

A total of 286 adults were recruited from several community centers in the province of Granada, Spain. The final sample consisted of 169 individuals. The sample of individuals with ID comprised 90 adults (48 men and 42 women) with an average age of 39.86 years. These participants were recruited from four centers for people with intellectual disabilities. The sample of individuals without ID comprised 79 adults (37 men and 43 women) from two municipal community centers, with an average age of 36.95 years.

The inclusion criteria for both groups were: over 18 years of age and voluntary participation. The additional criterion for the group of individuals with ID was a diagnosis of moderate ID. The diagnosis was made by the Andalusian Public Health System, which is the regional public health system to which the geographical area where the study was conducted belongs. The participants were screened by checking the medical reports available at the center where the participants were recruited. The exclusion criteria for both groups were: (i) severe behavioral disorders having an impact on the evaluation process (determined by Maladaptive Behavior Scale—MABS) [19], (ii) severe mental disorder, based on psychiatric evaluation records, (iii) severe language impairment, and (iv) severe cognitive impairment, determined by a score of ≥24 points in the Lobo Mini-Mental State Examination. Potential participants with language alterations, both in comprehension and expression, having a severe cognitive impairment or a mother tongue other than Spanish were excluded to ensure the correct administration of questionnaires.

### 2.3. Evaluation System

The evaluation sessions were conducted in a well-lit room located outside the occupational workshop, in the case of individuals with ID, and in the workshops held in the community centers occupied by subjects in the group of people without ID. Each session was divided into two parts. In the initial part of the session, participants were provided with an explanation of the study’s objectives and purpose. They were also informed of their right to withdraw from the study at any point as per their preference. During the latter segment of the session, lasting approximately 40–45 min, the assessment tools were administered by a researcher endowed with extensive expertise in individuals with ID. The instruments used in the present study were in a language-free version making them more suitable in cross-cultural and special needs contexts. The researchers were trained to ensure the consistency and integrity of data collection. An *ad hoc* sociodemographic questionnaire was used to collect information on sex, age, educational level, years of education, and literacy skills. In addition, we register clinical data such as manual dexterity and the presence of motor or sensory/perceptual disturbances by asking the participant or legal guardian, and by checking the medical reports. The executive functions and the ability to perform activities of daily living were evaluated by the following instruments.

The Lawton and Brody Scale was used to evaluate the ability to perform IADLs and the need for support in their performance [20,21]. This scale consists of eight items that measure functioning in use of the telephone, shopping, food preparation, housekeeping, laundry, mode of transportation, responsibility for medication, and handling finances. Total scores range from 0 (maximum dysfunction) to 8 points (total functionality) [20,21]. The scale was used in two complementary formats simultaneously: (i) an informant-based questionnaire completed by the occupational therapist working in the institution with the criterion of having known the person for at least three months before the evaluation in terms of the ability to perform the activity; (ii) observation of the level of function during performance of these activities. Observing the ability to perform IADLs is crucial in occupational therapy in order to determine an individual’s level of functioning. This scale has been validated for the Spanish population, showing good reliability and validity and obtaining a Cronbach’s alpha coefficient of 0.70 [22]. Currently, there is no other scale or instrument within the field of occupational therapy that is validated for the adult Spanish population to evaluate the level of functioning in IADLs.

Global executive control was evaluated using the total score of the INECO Frontal Screening (IFS) test in its Spanish version. The total score of the IFS test is 30 points, with the higher score indicating better executive functioning. On the other hand, this test consists of various domains of executive function: (i) motor programming, where the subject has to perform a sequence of hand movements; (ii) conflicting instructions, where the subject performs a sequence of movements that are the opposite of those performed by the evaluator; (iii) motor inhibitory control by go-no-go task, where the subject has to repeat the same sequence of movements performed by the evaluator; (iv) temporary cognitive information processing by backward digit span, where the subject must repeat a string of digits in reverse order; (v) verbal working memory, in which the subject is asked to list the months of the year in reverse order; (vi) spatial working memory, in which the evaluator presents the subject with four cubes and points at them in a given sequence. The subject is then asked to repeat the sequence in reverse order; (vii) reasoning task, in which three proverbs are read to the subjects, and they are asked to explain their meaning; (viii) verbal inhibitory control, which measures the subject’s capacity to inhibit an expected response. This test also estimates overall working memory, which is the total of the scores of the verbal and spatial working memory subtests. The IFS has been validated for Spanish population, with a Cronbach’s alpha of 0.80 [23].

Abstraction was evaluated using the similarities subtest from the fourth edition of the Wechsler Adult Intelligence Scale (WAIS-IV). This subscale comprises 18 items, in which the individual is presented with two apparently unrelated words and must identify their common characteristic. The total score ranges from 0 to 36 points, with a high score indicating a high level of abstraction [24]. This test has been validated for Spanish population, with a Cronbach’s alpha of 0.79 [11].

Mental processing was evaluated by the semantic verbal fluency test. This test consists of eliciting spontaneous words under a restrictive condition using category, or semantic, fluency. Subjects are asked to name as many items belonging to a certain category, in our case animals, as they can within 1 min. This test measures perseverations, intrusions, spelling errors, and paraphrases enunciated by the subject during the 1 min test duration. For this study, the maximum number of words verbalized without error was recorded in both groups [25,26].

Action planning ability was evaluated by using the test ‘key search’ from the Behavioural Assessment of the Dysexecutive Syndrome (BADS) battery. In this test, subjects have 95 s to draw with a pencil the path they would follow to find a key in an area delimited on the paper, starting from a pre-determined point. The maximum score is 16 points, with the higher score indicating greater planning capacity. The scores of this subtest were calculated for inclusion in the statistical analyses [27].

### 2.4. Procedures

The study took place between July 2019 and May 2020. Permission for recruitment within each participating center was granted by its respective directors. A written document detailing the study’s procedures and objectives was provided to the individuals overseeing each center. Subsequently, the researchers visited each center to acquaint themselves with the monitors and users, observe the facilities, and directly elucidate the study to the personnel responsible for each participant. Both study cohorts were sourced from each center using a consecutive sampling approach. This methodology involves enrolling participants sequentially as they become available, ensuring a continuous influx of participants into the study. The study’s particulars, aims, and procedures were comprehensively communicated to each potential participant and/or their legal guardian. Consent for participation was obtained in writing after explaining the study details. The informed consent package encompassed the participant’s information sheet, a register of informed consent forms, and a revocation letter. This protocol adheres to the precepts and principles outlined in the Declaration of Helsinki. The local Ethics Committee of CEI-Granada (Granada-Spain) approved the protocol of this study.

### 2.5. Data Analysis

The statistical analyses were performed on SPSS (Version 21.0). Descriptive data were presented as mean and standard deviation, in the case of continuous variables, and as a frequency in the case of categorical variables. Variables were assessed for normality through the Kolmogorov-Smirnov test (*p* > 0.05) or visual inspection of histograms. The group of subjects with ID was divided into two groups (dependency for IADLs vs. independent for IADLs) using the median of the sample in the Lawton and Brody scores (4 points). This dichotomization coincided in turn with one of the possible interpretations of the test scores that divides the individuals’ scores in dependency vs. independence for IADLs [28]. The categorical sociodemographic and clinical characteristics of the subjects were compared using the chi-square test. The student’s t for independent samples was used to compare age and test scores between groups (ID vs. controls) and between dependent vs. independent individuals. The equality of variance was analyzed using the Levene test (*p* > 0.05). We calculated the effect size (Cohen’s d) for variables that showed statistically significant differences in order to determine the magnitude of the differences between groups. The Pearson and Spearman bivariate correlation tests were used to determine the possible correlation between the degree of functioning in IADLs (Lawton and Brody test) and the remaining variables (sociodemographic, clinical, and executive functions). A linear regression analysis using a Stepwise method was performed to determine which variables predicted functioning in IADLs in the group of subjects with ID. The scores on the Lawton and Brody Scale were included as dependent variables, and variables that correlated significantly with this test were included as explanatory variables. Any explanatory variables that showed collinearity with each other, and that did not meet the assumptions of the regression model, were excluded. Statistical significance was set at *p* < 0.05 in all cases.

## 3. Results

**Figure 1:**
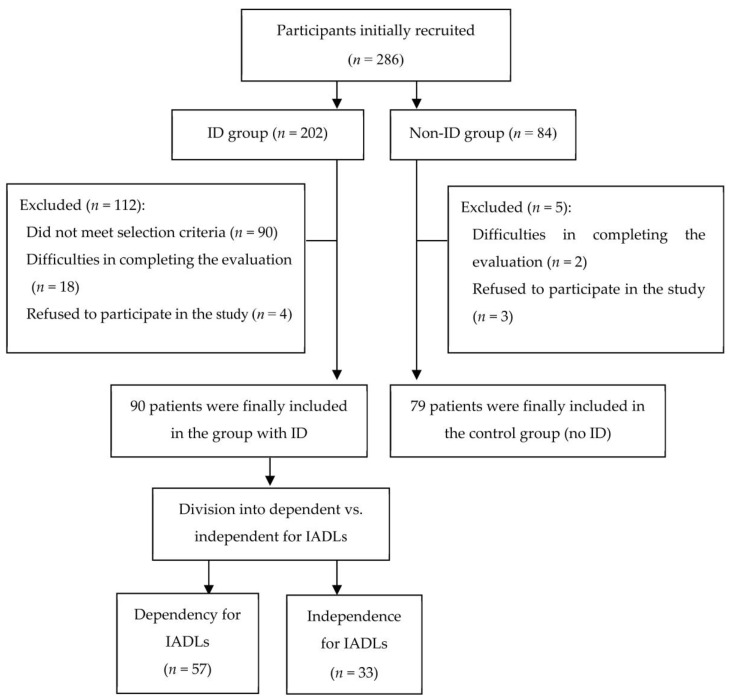
Flow diagram of subjects who participated in the study according to STROBE statement.

A total of 286 adults were recruited from several community centers in the province of Granada, Spain. After applying the selection criteria, a sample of 90 individuals with ID and 79 individuals without ID were included. In the group of people with ID, 48 (53.3%) were men and 42 (46.7%) women, with a mean (SD) age of 39.86 (11.31) years. In the group of people without ID, 37 (46.8%) were men and 43 (53.2%) women, with an average (SD) age of 36.95 years. Figure 1 shows the flow diagram of subjects who participated in the study according to the STROBE statement.

The sample with ID was divided into 2 groups, based on their degree of dependency for IADLs: dependent (*n* = 57) and independent (*n* = 33). In the ID group, 63.33% were dependent for IADLs, and 36.67% were independent for these activities. The sociodemographic and clinical characteristics and the comparison between the group with and without ID are shown in Table 1.

Our results showed statistically significant differences between the two groups (with and without ID) in mean scores for the Lawton and Brody scale, the similarities subtest, the IFS test, semantic verbal fluency, and the key test (*p* < 0.001). The calculation of Cohen’s d values (difference of means between groups) showed a moderate to large effect size between groups (minimum d = 0.653, maximum d = 3.064). The mean and SD of the results of these measurement variables for participants with and without ID, together with the effect sizes (difference of means between groups), are shown in Table 2.

Statistically significant differences in scores in all the executive function tests (*p* < 0.05) were also observed between groups with dependency and independence for IADLs. The effect sizes were moderate to large, with a minimum d value of 0.506 and a maximum d of 2.516. These results are shown in detail in Table 3.

In the group of institutionalized individuals with moderate ID, bivariate tests showed a direct correlation between the degree of IADL functioning and scores for the similarities subtest (*p* < 0.001), the overall score, and the scores obtained for different dimensions of the IFS test (*p* < 0.05), verbal semantic fluency (*p* < 0.001), and the key test (*p* < 0.001) (Table 4).

Linear regression analysis, meanwhile, showed that global executive control (total score on the IFS test) and the key test scores predicted the degree of IADL performance (total scores on the Lawton and Brody scale), predicting 81% of the total variance (R^2^ = 0.806) in the ability to perform these activities for the group with ID. The linear regression model is presented in Table 5.

## 4. Discussion

The first objective of this study was to evaluate the possible difference in the ability to perform IADLs and executive functioning between individuals with and without ID. In terms of IADLs performance, the results show that these individuals have moderate-large lower levels of functioning in IADLs compared to the sample of individuals without ID. The possible explanations for this difference are that: (i) adults with ID may experience greater difficulty in performing activities that require more complex cognitive processing and interaction with their physical and social environment. These activities involve a higher level of planning, inhibitory control in social situations, problem solving, and even memory processes [3]; (ii) the structured care and support provided by staff in the institution could limit subjects’ opportunities to participate in certain activities and prevent them from learning and performing such tasks. IADLs such as mobility in the community, shopping, bank transfers, or caring for others are usually planned and resolved by the institution. Institutionalized care could be eroding any such skills acquired at some point in their lives, because of disuse; (iii) individuals with ID presenting functional impairment from childhood may have elicited paternalistic attitudes toward them. The paternalist behaviors may have persisted into adulthood, thus limiting their chances of exploring and successfully performing daily activities; (iv) these behaviors could limit their capacity to develop and engage in certain roles such as parent, worker, or volunteer. These roles are usually vehicles for maintaining a satisfactory level of participation in the activity.

On the other hand, concerning executive functioning of individuals with ID compared with individuals without ID, exhibited poorer performance in abstraction, working memory, motor programming, verbal and motor inhibitory control, mental processing, cognitive flexibility, and action planning ability. The magnitude of these differences ranged from moderate to high for all variables. A history of low participation in daily tasks and activities could affect the acquisition of higher cognitive processes. In turn, low daily participation in activities could erode existing skills in these functions as a result of disuse [29,30]. Several studies have also found impairment of executive functions in this population [8,31,32,33]. One study [8] observed impairment of divided attention and verbal fluency after a 5-year follow-up of individuals with Down syndrome; however, in line with other studies, no impairment was observed in planning ability [8,34]. Similarly, another study [32] has reported that this population appears to experience difficulties in all dimensions of executive function. In line with our results, other researchers [31] have also observed a lower level of executive function performance and functionality in people with Down syndrome compared with a control group. More specifically, impairment has been observed in planning, spatial working memory, and verbal fluency, among other cognitive functions, using the same tests as those used in our study [33]. However, while previous research identified impairments in various cognitive functions in ID, including executive functions, the relationship between executive functioning and the performance of daily activities was not explored in those studies.

Regarding the second objective of the study, the degree of function-dysfunction in IADLs was directly related to all executive functions, insofar as poor performance of these functions was associated with greater dysfunction in IADLs. The global executive control and the action planning abilities predicted 81% of the explained variance of IADLs performance. Therefore, it could be argued that a decrease in executive function performance influences the degree of function in complex activities of daily living, or vice versa. However, we cannot confirm the directionality of this association, since our data were collected transversally. One explanation for this correlation is that higher cognitive functions are involved in processes that govern and monitor human behavior and conduct, enabling each person to interact with the world around him or her. These findings are in line with previous results; however, these latter have been observed in different populations, such as individuals with Alzheimer’s disease [29], elderly people living in the community [35], or individuals with acquired frontal lobe injury [12]. One study [29] found a significant correlation between executive dysfunction and functioning in IADLs. Another [30] found that psychomotor speed and mental flexibility are indicators of a decrease in functional capacity. However, other authors [12] have shown that slow thinking, impairment of selective attention (increased sensitivity to interference), and impairment of prospective memory may be the main predictors of performance of IADLs in individuals with frontal lesions, excluding sequencing, action planning ability, or mental flexibility. Hence, the present study contributes to the existing understanding by clarifying an association between executive functions and IADLs in the particular demographic being studied. The findings emphasize the complex interaction between executive functions and daily performance in people with ID.

### 4.1. Limitations

This study presents limitations. First, the transversal design prevented us from establishing a cause-effect relationship between the variables studied. Second, the sample was selected consecutively, which could affect the representativeness or external validity of the results with respect to the general population of institutionalized individuals with moderate ID. Thirdly, some authors contend that comparative groups should be evenly matched in terms of intellectual coefficient (mental age). However, this factor may be less important than chronological age in sample selection, since both groups will have had the same opportunity to accumulate life experiences or life episodes. Finally, another limitation of this study may be the absence of validated and reliable performance-based executive function measures in Spanish. While the current findings suggest the potential utility of such measures in understanding the relationship between executive functions and IADLs, there are currently no available instruments in Spanish that meet the required standards of validity and reliability, to the best of our knowledge.

### 4.2. Future Research

Longitudinal studies ought to be carefully designed to establish causality, or alternatively, studies employing larger and more diverse samples should be undertaken to enhance the generalizability of the findings. Therefore, studies with larger and more diverse samples are necessary to extend and validate these results to broader populations. Future research should also focus on developing and validating appropriate performance-based EF measures in Spanish to advance our understanding of this important clinical relationship. On the other hand, future studies could determine subjects’ IQ in order to compare chronological and mental age in relation to their executive functions and ability to perform activities of daily living. Finally, further knowledge is required about the degree of tailored and personalized support that these people need within a developmental approach. In this respect, the use of a compensatory-substitutive approach could be avoided when there may still be the possibility of framing the treatment within a developmental approach.

### 4.3. Implications for Clinical Practice

The findings of the study hold significant implications for rehabilitation, highlighting the importance of evidence-based intervention programs designed to address specific cognitive deficits. These programs aim to enhance functional independence and overall quality of life. In clinical practice, these implications have a range of potential applications. The insights from the findings offer valuable input for creating evidence-based healthcare and occupational therapy programs, covering educational, personal, and community skills, as well as cognitive stimulation interventions. Furthermore, the presented information has the potential to inspire innovative strategies tailored to meet individual needs. Lastly, the results can play a vital role in optimizing resource allocation and support for individuals with intellectual disabilities and their families. Furthermore, it is necessary to understand and generate support plans that enable the development of behavior compatible with the demands of the environment and consistent with the skills that need to be deployed in the community, such as impulse control and decision-making. Our results will also facilitate the design of strategies aimed at calibrating the real support that these people need. The more suitable and better tailored the support provided to a person with ID, the better the training of executive functions and the ability to perform IADLs.

On the other hand, the study results may represent a breakthrough in “activity analysis” models in occupational therapy, especially when working with individuals with intellectual disabilities. These findings suggest that individuals with intellectual disabilities exhibit poor performance across various cognitive domains, including abstraction, working memory, motor programming, inhibitory control, mental processing, cognitive flexibility, and planning. Activity analysis in occupational therapy involves evaluating how a person performs daily tasks and activities to identify problem areas and develop appropriate interventions. The study’s results indicate that individuals with intellectual disabilities may face specific challenges in performing these activities. Therefore, occupational therapists need to consider these executive functions when designing individualized treatment plans. These approaches could include training in strategies to improve working memory, inhibitory control, cognitive flexibility, and task organization. Additionally, social skills training and problem-solving techniques could be used to address difficulties in mental processing and abstraction. It is also essential to consider that each individual is unique, so occupational therapists must conduct comprehensive and personalized assessments to identify the specific needs of each person.

## 5. Conclusions

The study revealed that individuals with intellectual disabilities exhibited poor performance across various cognitive domains, including abstraction, working memory, motor programming, inhibitory control, mental processing, cognitive flexibility, and planning. Notably, the global executive control and the action planning abilities predicted 81% of the explained variance of IADLs performance. Therefore, institutionalized individuals with moderate ID may face challenges in performing IADLs, possibly linked to deficits in executive function performance. However, it is essential to acknowledge that the generalizability of these findings is limited due to the sample size and research design, which primarily focuses on contexts with similar characteristics to the geographical area of recruitment. At a clinical level, this information could contribute to the design and development of evidence-based interventional programs in this population that involve specific support strategies focused on the occupation to meet individual needs. In addition, the study results will facilitate the design of strategies aimed at calibrating the real support that these people need and can also be used to tailor therapeutic activities in order to develop a therapeutic tool that enhances executive functions.

## Figures and Tables

**Table 1:** Mean (SD) and frequency (%) for sociodemographic and clinical characteristics of participants with and without ID.

Sociodemographic and Clinical Characteristics	Group with ID Mean (SD)/ Frequency (%) *n* = 90	Group without ID Mean (SD)/ Frequency (%) *n* = 79	Student’s t/Chi-Square (χ^2^)
Age (years)	39.86 (11.31)	36.95 (14.59)	1.432
Sex			
Women	42 (46.7)	42 (53.2)	0.711
Men	48 (53.3)	37 (46.8)	
Level of education			
No schooling	4 (4.4)	0	51.535 **
Incomplete primary	24 (26.7)	4 (5.1)	
Primary	34 (37.8)	16 (20.3)	
Secondary	28 (31.1)	32 (40.5)	
Technical training	0	13 (16.5)	
University	0	14 (17.7)	
Years of education			
None	4 (4.4)	0	9.015 *
<5 years	9 (10.0)	4 (5.1)	
From 5 to 10 years	32 (35.6)	20 (25.3)	
>10 years	45 (50.0)	55 (69.6)	
Literacy			
Does not read or write	23	0	58.081 **
Reads and writes (with difficulty)	30	3	
Reads and writes (fluently)	37	76	
Handedness			
Right-handed	76	68 (86.1)	3.027
Left-handed	12	6 (7.6)	
Ambidextrous	2	5 (6.3)	
Motor disorders			
Yes	8	0	7.371 *
No	82	79 (100)	
Sensory disorders			
Yes	1	0 (0)	0.883
No	89	79 (100)	
Corrected visual disturbances			
Yes	8	0 (0)	7.371 *
No	82	79 (100)	
Corrected auditory disturbances			
Yes	0 (0)	0 (0)	-
No	90 (100)	79 (100)	

* *p* < 0.05; ** *p* < 0.001.

**Table 2:** Mean (standard deviation) and differences between groups with and without intellectual disability in performing instrumental activities of daily living and executive functions (*n* = 169).

Variable	Group with ID Mean (SD)/ *n* = 90	Group without ID Mean (SD)/ *n* = 79	Student’s *t*	Cohen’s *d*
Lawton and Brody scale	3.74 (2.20)	8 (0.00)	18.343 **	2.738
Global executive control	8.94 (8.18)	24.58 (3.32)	10.441 **	2.505
Motor programming	1.34 (1.28)	2.69 (0.64)	5.615 **	1.334
Conflicting instructions	0.94 (1.21)	2.86 (0.49)	8.630 **	2.079
Motor inhibitory control	1.09 (1.30)	2.73 (0.75)	6.625 **	1.545
Information processing	0.61 (1.00)	3.03 (1.52)	9.570 **	1.881
Verbal working memory	0.37 (0.70)	1.86 (0.46)	10.894 **	2.515
Spatial working memory	0.75 (1.11)	3.00 (0.90)	10.105 **	2.226
Reasoning	0.51 (0.90)	2.72 (0.48)	13.109 **	3.064
Verbal inhibitory control	3.31 (4.27)	5.70 (0.62)	3.132 *	0.783
General working memory	1.36 (1.96)	6.03 (1.91)	11.393 **	2.413
Abstraction	7.30 (5.84)	18.98 (6.33)	10.214 **	0.653
Mental processing	9.40 (7.36)	26.00 (7.94)	9.190 **	2.168
Action planning ability	2.81 (3.52)	9.98 (3.68)	8.441 **	1.991

IFS: INECO Frontal Screening; WM: Working memory. * *p* < 0.05; ** *p* < 0.001.

**Table 3:** Mean (standard deviation) and differences between groups with dependency and independence for executive functions in institutionalized individuals with moderate intellectual disability.

Variable	IADLs Dependency Mean (SD)/ *n* = 57	IADLs Independence Mean (SD)/ *n* = 33	Student’s *t*	Cohen’s *d*
Global executive control	3.95 (4.07)	17.25 (6.27)	−7.298 **	2.516
Motor programming	0.75 (1.16)	2.33 (0.78)	−4.171 **	1.598
Conflicting instructions	0.40 (0.88)	1.83 (1.19)	−3.895 *	1.366
Motor inhibitory control	0.45 (0.76)	2.17 (1.34)	−4.071 *	1.578
Information processing	0.10 (0.45)	1.46 (1.12)	−4.023 *	1.593
Verbal working memory	0.15 (0.49)	0.75 (0.87)	−2.199	0.849
Spatial working memory	0.20 (0.52)	1.67 (1.23)	−3.921 *	1.556
Reasoning	0.10 (0.31)	1.21 (1.12)	−3.361 *	1.237
Verbal inhibitory control	1.80 (1.61)	5.83 (5.99)	−2.870 *	0.918
General working memory	0.30 (0.80)	3.13 (2.07)	−4.532 *	1.803
Abstraction	6.14 (5.44)	9.06(6.08)	−2.253 *	0.506
Mental processing	5.80 (4.41)	15.42 (7.48)	−4.598 **	1.566
Action planning ability	1.00 (2.13)	5.83 (3.35)	−5.007 **	1.720

IADL: instrumental activities of daily living; IFS: INECO Frontal Screening; SD: standard deviation; WM: Working memory. * *p* < 0.05. ** *p* < 0.001.

**Table 4:** Correlation between the capacity to perform instrumental activities of daily living and executive functions in institutionalized individuals with moderate intellectual disability (*n* = 90).

Variable	Pearson Correlation (*r*)
Age (years)	−0.108
Overall executive function	0.854 **
Motor programming	0.596 **
Conflicting instructions	0.577 *
Motor inhibitory control	0.639 **
Information processing	0.610 **
Verbal working memory	0.540 *
Spatial working memory	0.649 **
Reasoning	0.677 **
Verbal inhibitory control	0.554 *
General working memory	0.679 **
Abstraction	0.486 **
Mental processing	0.628 **
Action planning ability	0.731 **

IFS: INECO Frontal Screening; WM: Working memory. * *p* < 0.05; ** *p* < 0.001.

**Table 5:** Linear regression model of executive functions as predictors of IADLs performance in institutionalized individuals with moderate intellectual disability.

Explanatory Variables	Lawton and Brody Scale (R^2^ = 0.806)					
	B	95% CI		β	SE	*p*-Value
		Lower Limit	Upper Limit			
IFS test (total score)	0.228	0.155	0.301	0.649	0.036	<0.001
Key test	0.281	0.111	0.450	0.344	0.083	0.002

R^2^, regression coefficient of determination; B, regression coefficient; CI, confidence interval; β, adjusted coefficient from multiple linear regression analysis; SE coefficient standard error.

## Data Availability

The data could be requested by the scientific community in the ethical terms to be determined.
